# Available evidence of antibiotic resistance from extended-spectrum β-lactamase-producing Enterobacteriaceae in paediatric patients in 20 countries: a systematic review and meta-analysis

**DOI:** 10.2471/BLT.18.225698

**Published:** 2019-05-14

**Authors:** Yanhong Jessika Hu, Anju Ogyu, Benjamin J Cowling, Keiji Fukuda, Herbert H Pang

**Affiliations:** a School of Public Health, Patrick Manson Building (North Wing), 7 Sassoon Road, Li Ka Shing Faculty of Medicine, University of Hong Kong, Hong Kong Special Administrative Region, China.

## Abstract

**Objective:**

To make a systematic review of risk factors, outcomes and prevalence of extended-spectrum β-lactamase-associated infection in children and young adults in South-East Asia and the Western Pacific.

**Methods:**

Up to June 2018 we searched online databases for published studies of infection with extended-spectrum β-lactamase-producing Enterobacteriaceae in individuals aged 0–21 years. We included case–control, cohort, cross-sectional and observational studies reporting patients positive and negative for these organisms. For the meta-analysis we used random-effects modelling of risk factors and outcomes for infection, and meta-regression for analysis of subgroups. We mapped the prevalence of these infections in 20 countries and areas using available surveillance data.

**Findings:**

Of 6665 articles scanned, we included 40 studies from 11 countries and areas in the meta-analysis. The pooled studies included 2411 samples testing positive and 2874 negative. A higher risk of infection with extended-spectrum β-lactamase-producing bacteria was associated with previous hospital care, notably intensive care unit stays (pooled odds ratio, OR: 6.5; 95% confidence interval, CI: 3.04 to 13.73); antibiotic exposure (OR: 4.8; 95% CI: 2.25 to 10.27); and certain co-existing conditions. Empirical antibiotic therapy was protective against infection (OR: 0.29; 95% CI: 0.11 to 0.79). Infected patients had longer hospital stays (26 days; 95% CI: 12.81 to 38.89) and higher risk of death (OR: 3.2; 95% CI: 1.82 to 5.80). The population prevalence of infection was high in these regions and surveillance data for children were scarce.

**Conclusion:**

Antibiotic stewardship policies to prevent infection and encourage appropriate treatment are needed in South-East Asia and the Western Pacific.

## Résumé

Objectif Réaliser une revue systématique des facteurs de risque, des conséquences et de la prévalence des infections à bêta-lactamases à spectre élargi chez des enfants et des jeunes adultes dans les régions de l'Asie du Sud-Est et du Pacifique occidental de l'Organisation mondiale de la Santé (OMS).

Méthodes Jusqu’en juin 2018, nous avons consulté des bases de données en ligne à la recherche d'études publiées portant sur des infections par des entérobactéries productrices de bêta-lactamases à spectre élargi chez des individus âgés de 0 à 21 ans. Nous avons inclus des études cas/témoins, de cohortes, transversales et d'observation rendant compte de patients positifs et négatifs à l'égard de ces organismes. Pour la méta-analyse, nous avons utilisé une modélisation à effets aléatoires des facteurs de risque et des conséquences associés aux infections, et une méta-régression pour l'analyse des sous-groupes. Nous avons analysé la prévalence de ces infections dans 20 pays et régions à l'aide des données de surveillance disponibles.

Résultats Sur les 6665 articles parcourus, nous avons inclus 40 études de 11 pays et régions dans la méta-analyse. Prises ensemble, les études incluaient 2411 échantillons déclarés positifs et 2874 négatifs. Un risque plus élevé d'infection due à des bactéries productrices de bêta-lactamases à spectre élargi a été associé à de précédents soins hospitaliers, et en particulier à des séjours en unité de soins intensifs (RC regroupés: 6,5; IC à 95%: de 3,04 à 13,73); à une exposition aux antibiotiques (en particulier aux céphalosporines de troisième génération, CR: 4,8; IC à 95%: de 2,25 à 10,27); et à certaines affections concomitantes. Le traitement antibiotique empirique a eu un effet protecteur vis-à-vis des infections (CR: 0,29; IC à 95%: de 0,11 à 0,79). Les patients infectés se caractérisaient par des séjours hospitaliers plus longs (26 jours; IC à 95%: de 12,81 à 38,89) et un risque de décès plus élevé (CR: 3,2; IC à 95%: de 1,82 à 5,80). La prévalence de l'infection dans la population était élevée dans ces régions et les données de surveillance relatives aux enfants étaient alarmantes.

Conclusion Des politiques de gestion des antibiotiques pour prévenir les infections et encourager un traitement approprié sont nécessaires dans les pays et les régions de l'Asie du Sud-Est et du Pacifique occidental de l'OMS.

## Resumen

**Objetivo:**

Realizar una revisión sistemática de los factores de riesgo, los resultados y la prevalencia de la infección de amplio espectro asociada a la betalactamasa en niños y adultos jóvenes en las regiones de Asia Sudoriental y el Pacífico Occidental de la Organización Mundial de la Salud (OMS).

**Métodos:**

Hasta junio de 2018 se realizaron búsquedas en las bases de datos en línea de estudios publicados sobre infección con Enterobacteriáceas productoras de betalactamasas de amplio espectro en individuos de 0 a 21 años de edad. Se incluyeron estudios de casos y controles, de cohortes, transversales y observacionales que reportaron pacientes positivos y negativos para estos organismos. Para el metanálisis se utilizó la modelización de efectos aleatorios de los factores de riesgo y los resultados en cuanto a la infección, y la metarregresión para el análisis de los subgrupos. Mapeamos la prevalencia de estas infecciones en 20 países y regiones utilizando los datos de vigilancia disponibles.

**Resultados:**

De los 6 665 artículos examinados, se incluyeron 40 estudios de 11 países y regiones en el metanálisis. Los estudios agrupados incluyeron 2 411 muestras positivas y 2 874 negativas. Un mayor riesgo de infección con bacterias productoras de betalactamasas de amplio espectro se asoció con la atención hospitalaria previa, en particular las estancias en unidades de cuidados intensivos (OR agrupado: 6,5; IC del 95 %: 3,04 a 13,73); la exposición a los antibióticos (especialmente las cefalosporinas de tercera generación, OR: 4,8; IC del 95 %: 2,25 a 10,27); y ciertas enfermedades coexistentes. El tratamiento antibiótico empírico fue protector contra la infección (OR: 0,29; IC del 95 %: 0,11 a 0,79). Los pacientes infectados tuvieron estancias hospitalarias más prolongadas (26 días; IC del 95 %: 12,81 a 38,89) y mayor riesgo de muerte (OR: 3,2; IC del 95 %: 1,82 a 5,80). La prevalencia de la infección en la población era alta en estas regiones y los datos de vigilancia para los niños eran alarmantes.

**Conclusión:**

Se necesitan políticas de gestión de los antibióticos para prevenir la infección y fomentar el tratamiento adecuado en los países y zonas de las regiones de Asia Sudoriental y el Pacífico Occidental de la OMS.

## ملخص

**الغرض:**

إجراء مراجعة منهجية عوامل خطر ونتائج وانتشار طيف العدوى ممتدة النطاق المرتبطة ببيتا لاكتاميز الممتد في مناطق جنوب شرق آسيا وغرب المحيط الهادي التابعة لمنظمة الصحة العالمية.

**الطريقة:**

قمنا بالبحث في قواعد بيانات الإنترنت على الدراسات المنشورة حتى يونيو 2018 حول الإصابة بالطفيليات المعوية المنتجة ممتدة النطاق المنتجة بلبيتا لاكتاميز في الأفراد من الولادة وحتى 21 عاماً. قم بتضمين دراسات مراقبة الحالات، والمجموعات، والدارسات متعددة القطاعات والدراسات القائمة على الملاحظة، والتي تعد تقارير حول مدى إيجابية أو سلبية المرضى لهذه الكائنات. بالنسبة للتحليل التلوي، قمنا باستخدام وضع النماذج للآثار العشوائية لعوامل الخطر ونتائج العدوى، والانحدار التلوي لتحليل المجموعات الفرعية. قمنا بوضع خرائط لانتشار هذه الإصابات في 20 دولة ومنطقة باستخدام بيانات المراقبة المتاحة.

**النتائج:**

من إجمالي 6665 مقالاً تم فحصها، قمنا بتضمين 40 دراسة من 11 دولة ومنطقة في التحليل التلوي. شملت الدراسات المجمعة 2411 عينة اختبار إيجابية و 2874 سلبية. ارتبط خطر العدوى الأعلى بالنطاق الممتد الموسع للبكتيريا المنتجة للبيتا لاكتاميز، بالرعاية السابقة بالمستشفيات، وخاصةً الإقامة في وحدة العناية المركزة (نسبة احتمال مجمعة: 6.5؛ ‏بفاصل ثقة 95%: 3.04 إلى 13.7)؛ التعرض للمضادات الحيوية (وخاصةً سيفالوسبورين من الجيل الثالث، نسبة احتمال: 4.8؛ ‏بفاصل ثقة 95%: 2.25 إلى 10.27)؛ بعض الشروط المتواجدة معاً. كان العلاج بالمضادات الحيوية التجريبية واقياً من العدوى (نسبة احتمال: 0.29؛ ‏بفاصل ثقة 95%: 0.11 إلى 0.79). أقام المرضى المصابون لفترات أطول في المستشفى (26 يوماً؛ بفاصل ثقة 95%: 12.81 إلى 38.89) ومخاطر أعلى للوفاة (نسبة احتمال: 3.2؛ ‏بفاصل ثقة 95%: 1.82 إلى 5.80). كان انتشار العدوى بين السكان مرتفعًا في هذه المناطق وكانت بيانات المراقبة الخاصة بالأطفال مفزعة.

**الاستنتاج:**

هناك حاجة إلى سياسات رعاية للمضادات الحيوية لمنع الإصابة وتشجيع العلاج المناسب في البلدان ومناطق جنوب شرق آسيا وغرب المحيط الهادي التابعة لمنظمة الصحة العالمية.

## 摘要

**目的:**

对世卫组织东南亚和西太平洋区域儿童和青年人的超广谱 β内酰胺酶相关感染的风险因素、结果和感染率情况进行了系统审查。

**方法:**

截至 2018 年 6 月，我们于在线数据库中搜索了 0-21 岁个体中超广谱 ββ内酰胺酶肠杆菌科感染相关的已发表的研究。我们包含了报告患者此类组织的阳性和阴性状态的病例对照、队列研究、横断面研究和观察性研究。对于荟萃分析，我们采用风险因素和感染结果的随机效应进行建模，并分析亚组元回归。我们使用现有监测数据，绘制了 20 国和地区感染率情况图。

**结果:**

扫描的 6665 篇文章中，我们在荟萃分析中纳入了来自 11** **个国家和地区的** **40项研究。汇总研究包括2411** **个检测阳性样本和** **2874个检测阴性样本。超广谱ββ 内酰胺酶细菌感染风险较高，与先前住院治疗有关，特别是重症监护室住院（合并OR：6.5，95％ 置信区间，CI：3.04 至 13.73）；抗生素暴露（特别是第三代头孢菌素，OR：4.8，95％ 置信区间，CI：2.25至10.27）；和某些共存疾病。实证抗生素治疗可预防感染（OR：0.29，95％ 置信区间，CI：0.11 至 0.79)。感染患者住院时间较长（26 天；95％ 置信区间，CI：12.81 至 38.89），并伴有更高的死亡风险（OR：3.2，95％ 置信区间，CI：1.82 至 5.80)。这些地区人口感染率极高且儿童监测数据骇人。

**结论:**

世卫组织东南亚和西太平洋区域的国家和地区需采取抗生素管理政策，用以预防感染并鼓励适当治疗。****

## Резюме

**Цель:**

Выполнить системный обзор факторов риска, исходов и распространенности инфекций, вызванных микроорганизмами, продуцирующими β-лактамазу расширенного спектра, у пациентов детского и юношеского возраста в Юго-Восточной Азии и Западно-Тихоокеанском регионе (по классификации ВОЗ).

**Методы:**

Авторы изучили сетевые базы данных на предмет статей, датированных до июня 2018 года и посвященных исследованиям инфекций, вызванных продуцирующими β-лактамазу энтеробактериями, у пациентов в возрасте от 0 до 21 года. В рассмотрение включались обсервационные, межгрупповые, когортные исследования и исследования типа «случай-контроль», в которых сообщалось о пациентах с положительными и отрицательными результатами анализов на наличие указанных организмов. Для метаанализа использовалось моделирование факторов риска и исходов инфекции со случайными эффектами. Анализ подгрупп проводился с применением метарегрессии. Была составлена карта распространенности таких инфекций в 20 странах и регионах с использованием имеющихся данных санитарного надзора.

**Результаты:**

Из 6665 просмотренных статей в метаанализ были включены 40 исследований, охватывающих 11 стран и регионов. В общей сложности исследования включали 2411 положительных и 2874 отрицательных образца. Более высокий риск инфицирования бактериями, вырабатывающими β-лактамазу расширенного спектра, отмечался у пациентов, ранее получавших лечение в больничных условиях, особенно этот эффект был заметен для пациентов ОИТ (обобщенное ОШ: 6,5; 95%-й ДИ: от 3,04 до 13,73), у тех, кто принимал антибиотики (особенно цефалоспорины третьего поколения, ОШ: 4,8; 95%-й ДИ: от 2,25 до 10,27), и при некоторых сопутствующих заболеваниях. Эмпирическое лечение антибиотиками имело защитный эффект в отношении инфекции (ОШ: 0,29; 95%-й ДИ: от 0,11 до 0,79). Инфицированные пациенты дольше оставались в больнице (26 дней, 95%-й ДИ: от 12,81 до 38,89) и имели более высокий риск смерти (ОШ: 3,2; 95%-й ДИ: от 1,82 до 5,80). Частота инфекции в популяции указанных регионов была высокой, а данные надзора среди детей — ужасающими.

**Вывод:**

В странах Юго-Восточной Азии и в Западно-Тихоокеанском регионе (по географической классификации ВОЗ) необходимо ввести политику ответственного назначения антибиотиков для предотвращения инфекции и поощрения правильного лечения.****

## Introduction

Antimicrobial resistance occurs when bacteria are no longer susceptible to the drugs used for treatment.^1^ Increasingly, there are fewer antimicrobial drugs available to effectively treat common as well as life-threatening infections. Annual deaths from untreatable infections may rise from estimated 700 000 in 2015 to 10 million by 2050 if antimicrobial resistance is not controlled.^2^ Common procedures such as surgery or cancer chemotherapy may become too dangerous to perform without effective antibiotics.

Extended-spectrum β-lactamases are enzymes that cause resistance to some of the most commonly used antibiotics,^3^ including all penicillins, cephalosporins and monobactams.^3^ Fortunately these enzymes have yet to confer resistance to carbapenems, making these drugs valuable for serious extended-spectrum β-lactamase-producing bacterial infections.^4^ However, there have been recent outbreaks of extended-spectrum β-lactamase-producing *Klebsiella* spp. with carbapenem resistance, resulting in extremely high rates of mortality.^5^^,^^6^ Within the already limited selection of antibiotics available to treat these infections, fewer are approved for use in children.^7^ Children are particularly vulnerable to bacterial infections compared with young adults, due to their immature immune systems.^8^^,^^9^

The World Health Organization (WHO) South-East Asia and Western Pacific Regions have over 4.3 billion of the world’s population of 7.7 billion, including two of the most populous countries with heavy consumption of antibiotics: China and India.^10^ Research suggests these regions have high antimicrobial resistance rates to extended-spectrum β-lactamase-producing bacteria in the paediatric population.^11^ Poor-quality antibiotics and unsupervised use are common across the Regions. The available studies provide an overall impression of the prevalence of antibiotic resistance in the Regions, but better evidence is needed about the risk factors and outcomes for children with these infections. We therefore aimed to make a systematic review and meta-analysis of the risk factors and outcomes of infection with extended-spectrum β-lactamase-producing Enterobacteriaceae in children and young adults in the South-East Asia and the Western Pacific. We also mapped the prevalence of extended-spectrum β-lactamase-associated infections in countries and areas of the Regions using the available surveillance data.

## Methods

### Meta-analysis

We conducted the meta-analysis in accordance with the *Cochrane handbook for systematic reviews of interventions*.^12^ All procedures followed Preferred Reporting Items for Systematic Reviews and Meta-Analyses guidelines.^13^ The study was registered in the PROSPERO International prospective register of systematic reviews (CRD42017069701).

#### Search strategy

We made a comprehensive search, without language limitation, of online databases for articles published from 1 January 1940 to 30 June 2018 (Box 1). Two researchers independently conducted the search and screened the titles, abstracts and full texts of the papers. We used a standardized, piloted data collection form to determine whether papers were appropriate for inclusion. The researchers applied the Newcastle–Ottawa scale to assess risk of bias in non-randomized studies.^14^ Studies scoring ≥ 5 and ≤ 8 were designated low risk of bias, ≥ 3 and ≤ 4 as moderate and ≤ 2 as high. We incorporated the quality assessment results into our sensitivity analysis using the Meta-analyses Of Observational Studies in Epidemiology checklist. Discrepancies at any stage of the analysis were resolved by consensus of the researchers.

Box 1 **Search strategy used in the systemic review of extended-spectrum β-lactamase-associated infection among children and young adults in South-East Asia and Western Pacific countries**

We searched online databases (Embase®, MEDLINE®, Cochrane Library, Web of Science, Scopus, OvidSP®, EBSCO), electronic abstract databases and references in published articles. For the prevalence study we also searched the grey literature, including the websites of the World Health Organization and the United States Centers for Disease Control, surveillance systems related to antimicrobial resistance, dissertations, conference reports and country reports. When more information about studies was needed we contacted authors or website administrators.

We used the following keywords:

extended-spectrum beta-lactamase OR extended-spectrum beta-lactamase OR ESBL* OR ESBLs OR ESBL-producing*

AND paediatric OR pediatric OR juvenile OR child OR children OR adolescence OR infant OR neonat* OR neonatal OR newborn OR nursery

AND Asia OR Asia Pacific OR South Asia OR The Western Pacific OR South-East Asia OR Australia OR Bangladesh OR Bhutan OR Brunei Darussalam OR Cambodia OR China OR Cook Islands OR Democratic People's Republic of Korea OR Fiji OR India OR Indonesia OR Japan OR Kiribati OR Lao People's Democratic Republic OR Malaysia OR Maldives OR Marshall Islands OR Federated States of Micronesia (Federated States of) OR Mongolia OR Myanmar OR Nauru OR Nepal OR New Zealand OR Niue OR Palau OR Papua New Guinea OR Philippines OR Republic of Korea OR Samoa OR Singapore OR Solomon Islands OR Sri Lanka OR Thailand OR Timor-Leste OR Tonga OR Tuvalu OR Vanuatu OR VietNam

#### Selection criteria

We included cohort, case–control and observational or cross-sectional studies. We defined the target population as children aged from birth to 21 years, according the American Academy of Paediatrics guidelines.^15^ We included studies that were conducted in the WHO South-East Asia and Western Pacific Regions and that recorded both positive and negative results of testing for extended-spectrum β-lactamase-producing bacteria.

#### Outcome measures

The principal outcome measure was patients’ infection status, defined by whether specimens obtained tested positive or negative for infection with extended-spectrum β-lactamase-producing bacteria. We analysed infection status by risk sub-groups: medical history in the 3 months before the infection (hospital stay, intensive care unit admission, surgery), exposure to invasive life support, antibiotic therapy and co-morbidities or underlying conditions. Other outcomes recorded were: hospital length of stay, mortality, persistent bacteraemia, antibiotic residence and duration of fever after antibiotic therapy.

#### Data synthesis and analysis

For the meta-analysis we pooled the data on number of isolates (four studies) or patients with isolates (37 studies) using a Mantel–Haenszel random-effects model to determine the risk of infection with extended-spectrum β-lactamase-producing bacteria.^16^ We calculated pooled odds ratio (OR) and 95% confidence intervals (CI) for dichotomous outcomes and weighted mean difference and 95% CI for continuous outcomes. All tests were two-tailed and *P* < 0.05 was considered statistically significant. If studies provided median and interquartile range, we made estimates of the mean and standardized deviation (SD).^17^

We assessed the heterogeneity of the studies using the *I^2^* statistic, which evaluates the consistency of study results. The cut-off for defining heterogeneity was *I^2^* > 50%.^18^ Our sensitivity analyses were based on sample size on the overall summary estimates.^19^ We evaluated whether this restricted analysis affected the magnitude, direction and statistical significance of the overall summary estimate. Additional sensitivity analyses assessed the different types of study designs, settings and risk of bias.

We carried out meta-regression to explore each potential factor contributing to heterogeneity between studies, such as study location, design, duration and setting, and patients’ age and diagnosis, for all included studies reporting mortality and persistent bacteraemia. We used funnel plots with Egger regression test to assess publication bias (*P* < 0.1). 

All statistical analyses were performed with R software, version 3.4.0 (R Foundation for Statistical Computing, Vienna, Austria), using the Meta and Metafor meta-analysis packages.

### Prevalence study

We obtained data on the prevalence of extended-spectrum β-lactamase-associated infection from the same studies included in the meta-analysis. We also made a search for other data sources in the published and grey literature (Box 1). We included data on children (ages 0–21 years), where available, and all age groups, if data for these ages were unavailable. We calculated percentage prevalence by the number of people or isolates testing positive for extended-spectrum β-lactamases out of the total population or isolates tested. For case–control studies, the overall prevalence rate was extracted instead. Numbers of cases and samples were extracted if stated by the source. Where population maps were provided in the source material, the average of the range were extracted as the prevalence in the country. We pooled the prevalence data by calculating the mean of the extracted data from all sources for each country.

## Results

### Meta-analysis

#### Study selection and characteristics

**Fig. 1:**
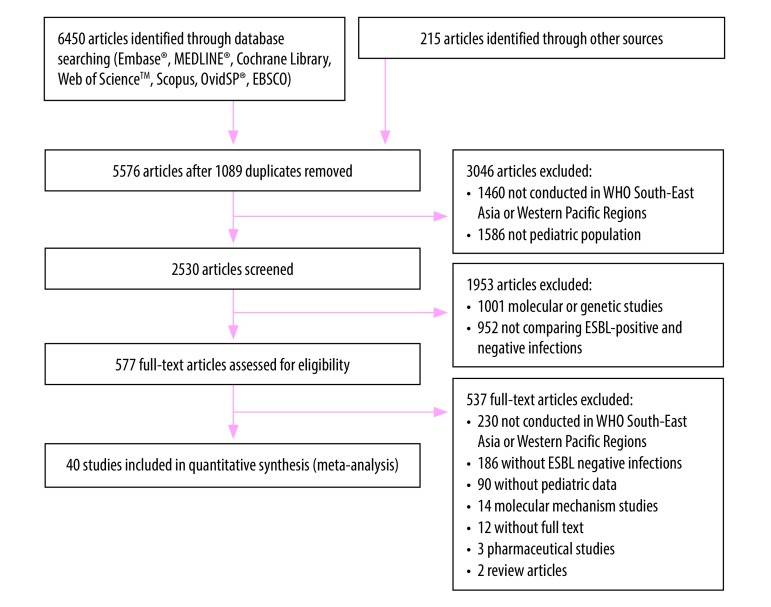
Flow diagram of the systematic review of extended-spectrum β-lactamase-associated infection among children and young adults in South-East Asia and Western Pacific countries

EBSL: extended-spectrum β-lactamase-producing bacteria; WHO: World Health Organization.

The database search yielded 6665 articles. We removed 1089 duplicates and excluded a further 3046 studies after screening titles and abstracts. After assessing the full text of 577 studies, we excluded 537. Screening of reference lists and conference abstracts yielded no additional studies. In total, 40 studies were included in the meta-analysis (Fig. 1). Three studies were reported in Chinese language, one study each in Korean and French, and the remaining 35 were in English. 

Overall, the 40 studies reported 46 960 bacterial isolates from 17 829 children providing samples. We pooled data from 2411 samples testing positive and 2874 testing negative for extended-spectrum β-lactamase-producing bacteria over the study period up to June 2018. The most common method of detection of bacterial phenotypes was agar disk diffusion in 32 studies. The study designs were 11 retrospective cohort studies, 14 prospective cohort studies, six observational studies, two cross-sectional studies and seven case–control studies. We found studies from 11 different countries and areas: Taiwan, China; India; Indonesia; Japan; Malaysia; Republic of Korea; Singapore; Sri Lanka; Thailand; and Viet Nam. In 15 studies the focus was specifically on neonates (< 28 days old), 15 studies were of age groups 0–21 years (excluding neonates), seven studies were of age 0–5 years (excluding neonates) and three studies did not specify the ages (Table 1; available at: http://www.who.int/bulletin/volumes/96/7/18-225698). 

**Table 1:** Characteristics of 40 studies included in the meta-analysis of extended-spectrum β-lactamase-associated infection among children and young adults in South-East Asia and Western Pacific countries, 2002–2018

Author	Country or area	Study dates	Study design	Study duration	Study setting	Diagnosis	Specimen site	Sample ages	No. of children	No. of samples		Prevalence of ESBL infection, %	No. of isolates or cultures	Bacterial species	ESBL detection method	Guidelines used
										ESBL-positive	ESBL-negative					
Kim et al., 2002^20^	Republic of Korea	Nov 1993–Dec 1998	Cohort	5 years	Community	Urinary tract infection	Urine	0–17 years	142	49	93	17	157	*Escherichia coli*, *Klebsiella pneumoniae*	Double disk diffusion	NCCLS, 2002
Jain et al., 2003^21^	India	1 year	Cohort	1 year	Hospital	Sepsis	Blood	Neonates	728	165	36	79	400	*E. coli*, *K. pneumoniae, Enterobacter* spp.	Double disk synergy test	CLSI, 2000 & NCCLS, date NS
Boo et al., 2005^22^	Malaysia	1996–Oct 2002	Case–control	7 years	Hospital	Sepsis	Various	Neonates	350	80	80	22	369	*K. pneumoniae*, *Enterobacter* spp.	Double disk diffusion	Ministry of Health of Malaysia, 2001
Chiu et al., 2005^23^	Taiwan, China	Jan 2001–Dec 2001	Cohort	1 year	Hospital	Nosocomial infection	Various	Neonates	76	34	42	44	76	*E. coli*, *K. pneumoniae,* KS	Double disk diffusion	NCCLS, 2001
Huang et al., 2007^24^	China	Jan 2000–Dec 2002	Cohort	3 years	Hospital	Nosocomial infection	Various	Neonates	39	22	17	56	2358	*E. coli*, *K. pneumoniae*	Double disk diffusion	NCCLS, 2000
Jain & Mondal, 2007^25^^,b^	India	Jan 2004–Dec 2005	Cohort	2 years	Hospital	Sepsis	Blood	Neonates	100	58	42	58	2995	*K. pneumoniae, Enterobacter* spp.	Double disk diffusion	NCCLS, 2003
Kuo et al., 2007^26^	Taiwan, China	Jan 2000–Oct 2005	Case–control	5 years 9 months	Hospital	Various	Various	Birth to NS	108	54	54	28	274	*K. pneumoniae *	Double disk diffusion	NCCLS, 2001
Lee et al., 2007^27^	Republic of Korea	Jan 1999–Dec 2005	Cohort	7 years	Hospital	Various	Various	NS	228	35	54	29	252	*E. coli*, *K. pneumoniae*	Double disk synergy test, Vitek-GNI card	CLSI, 2005
Sehgal et al., 2007^28^	India	April 2002–May 2003	Cohort	1 year	Hospital	Sepsis	Blood	Neonates	75	38	25	61	75	Multiple species^a^	Double disk diffusion	NCCLS, 2002
Bhattacharjee et al., 2008^29^^,b^	India	14 months	Cohort	1 year 2 months	Hospital	Sepsis	Blood	Neonates	243	26	58	32	243	Multiple species^a^	Double disk diffusion	CLSI, 2008
Anandan et al., 2009^30^^,b^	India	Jan 2003–Dec 2007	Cohort	5 years	Hospital	Sepsis	Blood	Neonates	94	68	26	72	8330	*E. coli*, *K. pneumoniae*	Not specify	CLSI, 2008
Kim et al., 2009^31^	Republic of Korea	Jan 2004–Apr 2009	Cohort	5 years 2 months	Community	Urinary tract infection	Urine	Children	854	32	83	17	681	*E. coli*, *K. pneumoniae*	Vitek 2 system	CLSI, date NS
Shakil et al., 2010^32^	India	Jan 2006–Feb 2007	Cohort	1 years	Hospital	Various	Various	Neonates	238	104	107	44	469	*E. coli*, *K. pneumoniae*	Double disk diffusion	CLSI, date NS
Gaurav et al., 2011^33^	India	May 2007–Apr 2008	Case–control	1 year	Hospital	Sepsis	Blood	Neonates	344	50	52	36	5116	*E. coli*, *K. pneumoniae*	Double disk diffusion	CLSI, date NS
Liu et al., 2011^34^	China	Feb 2009–Jan 2011	Cohort	2 years	Hospital	Lower respiratory tract infection	Sputum	< 3 years	242	94	148	39	242	Multiple species^a^	Double disk synergy test	CLSI, date NS
Wei et al., 2011^35^	China	Jan 2009–Dec 2009	Observational	1 year	Hospital	Lower respiratory tract infection	Sputum	< 1 year	272	144	128	53	1380	Multiple species^a^	Double disk synergy test	CLSI, 2009
Minami et al., 2012^36^	Japan	July 2011 (1 day)	Cross-sectional	1 day	Hospital	Various	Rectal	≤ 12 years	50	44	6	12	62	Multiple species^a^	Double disk synergy test	CLSI, 2008
Zheng et al., 2012^37^	China	2002–2008	Cohort	6 years	Hospital	Haematological malignancy	Blood	< 16 years	109	19	38	52	3264	*E. coli*, *K. pneumoniae*	Vitek 60 system	NCCLS, date NS
Vijayakanthi et al., 2013^38^	India	Dec 2009–Nov 2010	Cohort	1 year	Hospital	Sepsis	Various	Neonates	150	8	39	17	150	Multiple species^a^	Double disk diffusion	CLSI, date NS
Fan et al. 2014^39^	Taiwan, China	2002–2006	Case–control	4 years	Community	Urinary tract infection	Urine	< 15 years	312	104	208	33	6467	*E. coli*	Double disk diffusion	CLSI, 2007
Themphachana et al., 2014^40^	Thailand	Feb–Sep 2013	Observational	8 months	Hospital	Urinary tract infection	Urine	< 21 years	166	82	83	26	166	*E. coli*, *K. pneumoniae*	Double disk diffusion	CLSI, 2012
Young et al., 2014^41^	Singapore	Nov 2006–Feb 2007	Observational	3 months	Community	Various	Various	< 21 years	1006	69	124	4	1006	ESBL-producing Enterobacteriaceae, methicillin-resistant *Staphylococcus aureus*; vancomycin-resistant *Enterococcus* spp.	Double disk diffusion	CLSI, 2007
Zuo et al., 2014^42^	China	Jan–Dec 2013	Observational	1 year	Hospital	Lower respiratory tract infection	Sputum	1‒3 months	622	93	94	79	379	*E. coli*, *K. pneumoniae*	Kirby-Bauer disk diffusion	CLSI, 2012
Duong et al., 2015^43^	Viet Nam	Jul 2011–Nov 2012	Cohort	1 year 4 months	Hospital	Urinary tract infection	Various	3 months‒15 years	216	22	17	52	143	*E. coli*, *K. pneumoniae*	Double disk diffusion	CLSI, 2007
Han et al., 2015^17^^,c^	Republic of Korea	Apr 2009–Mar 2013	Cohort	4 years	Hospital	Neutropoenia (febrile)	Blood	< 20 years	61	21	40	34	61	*E. coli*, *K. pneumoniae*	Vitek 2 system	NS
Han et al., 2015^44^^,d^	Republic of Korea	Jan 2010–Dec 2014	Cohort	4 years	Hospital	Urinary tract infection	Urine	< 18 years	205	22	189	10	211	*E. coli*, *K. pneumoniae*	Vitek 2 system	NS
Nisha et al., 2015^45^	India	Nov 2012–Jan 2015	Cohort	3 years	Community	Urinary tract infection	Urine	≤ 18 years	385	159	226	41	385	*E. coli*	Kirby-Bauer disk diffusion	CLSI, date NS
Agarwal et al., 2016 ^46^^,b^	India	2009–2012	Cohort	4 years	Hospital	Diarrhoea	Stool	Young children	6339	23	98	19	6339	*E. coli*, *K. pneumoniae*	Vitek 2 system	CLSI, date NS
Amornchaicharoensuk, 2016^47^	Thailand	Jan 2010–Dec 2014	Cohort	5 years	Hospital	Urinary tract infection	Urine	0–15 years	117	19	69	16	117	*E. coli*, *K. pneumoniae*	Hospital laboratory	CLSI, date NS
Sharma et al., 2016^48^	India	Jan 2013–Aug 2014	Observational	1 year 7 months	Hospital	Sepsis	Blood	Neonates	1449	101	66	61	1449	Multiple species^a^	Double disk synergy test	NCCLS, date NS
Tsai et al., 2016^49^	Taiwan, China	Jan 2001–Dec 2012	Case–control	12 years	Hospital	Bacteraemia	Blood	Neonates	350	77	316	14	542	Multiple species^a^	Double disk synergy test	CLSI, 2012
Chen et al., 2017^50^	Taiwan, China	Jan 2004–Jul 2015	Cross-sectional	11 years	Hospital	Bacteraemia	Blood	Neonates	27	5	22	19	27	*E. coli*	Not specify	NS
He et al., 2017^51^	China	Mar 2011–Jun 2016	Cohort	4 years 3 months	Hospital	Lower respiratory tract infection	Sputum	1 month‒5 years	236	64	72	47	2360	*E. coli*, *K. pneumoniae*	Double disk synergy test	CLSI, date NS
Kim et al., 2017^52^	Republic of Korea	Jan 2010–Jun 2015	Cohort	5 years 5 months	Hospital	Bacteraemia	Blood	≤ 17 years	185	49	93	35	185	*E. coli*, *K. pneumoniae*	Double disk synergy test	NCCLS, 2001
Mandal et al., 2017^53^	India	Two consecutive year	Cohort	2 years	Community	Diarrhoea	Stool	0–60 months	633	72	119	38	633	*E. coli*	Modified Kirby-Bauer disk diffusion	CLSI, date NS
Nisha et al., 2017^54^	India	Nov 2012–Mar 2016	Cohort	4 years 5 months	Community	Urinary tract infection	Urine	3 months‒18 years	523	196	327	38	523	*E. coli*	Kirby-Bauer disk diffusion	CLSI, 2010
Tsai et al., 2017^55^	Taiwan, China	2010–2014	Observational	5 years	Hospital	Bacteraemia	Blood	< 3 years	41	14	27	34	41	*E. coli*	NS	NS
Bunjoungmanee et al., 2018^56^	Thailand	Jun 2016–May 2017	Case–control	1 year	Hospital & community	Urinary tract infection	Urine	1 month‒5 years	80	40	40	23	80	*E. coli*, *K. pneumoniae*	Double disk diffusion	CLSI, 2010
Kitagawa et al., 2018^57^	Indonesia and Japan	Jan–Nov 2014	Case–control	1 year	Hospital & community	Urinary tract infection	Urine	0–15 years	94	37	13	39	94	*E. coli*, *K. pneumoniae*	Double disk diffusion	CLSI, date NS
Weerasinghe et al., 2018^58^	Sri Lanka	Jan–April 2011	Cohort	3 months	Hospital	Various	Various	Neonates	50	18	8	36	50	*E. coli*, *K. pneumoniae*	Double disk diffusion	CLSI & CDC, 2011

CDC: Centers for Disease Control and Prevention; CLSI: Clinical and Laboratory Standards Institute; ESBL: extended-spectrum β-lactamase-producing bacteria; NCCLS: National Committee for Clinical Laboratory Standards; NS: not specified.

^a^ Multiple species included *Klebsiella pneumonia*; *Escherichia coli*; *Pseudomonas* spp.; *Acinetobacter* spp.; *Enterobacter* spp.; and *Citrobacter* spp.

^b^ Studies with data only on isolates; the remaining studies included data on patients and isolates.

^c^ Neutrpoenia study.

^d^ Urinary tract infection study.

#### Risk factors

The risk of infection with extended-spectrum β-lactamase-producing bacteria was significantly higher for patients whose medical history included intensive care unit admission (OR: 6.5; 95% CI: 3.04 to 13.73; *I^2^*: 65%; six studies), hospitalization (OR: 3.3; 95% CI: 1.95 to 5.57; *I^2^*: 80%; 11 studies) or surgery (OR: 2.3; 95% CI: 1.41 to 3.81; *I^2^*: 25%; six studies; Table 2).

**Table 2:** Pooled risk of extended-spectrum β-lactamase-associated infection among children and young adults in South-East Asia and Western Pacific countries by medical history and co-morbid conditions, 2002–2018

Subgroup	No. of studies	Total no. of patients	ESBL-positive, no.			ESBL-negative, no.		Pooled OR (95% CI)^a^	*I^2^*, %
			Events	Total		Events	Total		
**Received medical care in previous 3 months**									
Recent intensive care unit stay	6	1258	124	399		65	859	6.46 (3.04 to 13.73)	65
Recent hospitalization	11	2936	318	727		367	2209	3.30 (1.95 to 5.57)	80
Recent surgery	6	1178	58	433		37	745	2.32 (1.41 to 3.81)	8
Pre-infection hospitalization	3	223	NA	110		NA	113	11.42^b^ (−7.86 to 30.71)	99
**Diagnosis of co-morbid or underlying conditions**									
Bacteraemia	6	958	103	222		109	736	5.30 (3.64 to 7.72)	38
Lower respiratory tract infection	4	837	213	395		134	442	5.01 (3.50 to 7.19)	79
Recurrent urinary tract infection	11	2149	355	808		328	1341	2.01 (1.67 to 2.43)	90
Nosocomial infection	2	114	40	55		21	59	5.19 (2.23 to 12.07)	92
Various diagnoses	7	1772	229	545		339	1227	2.68 (2.06 to 3.48)	79
Sepsis	10	970	397	550		146	420	4.61 (3.34 to 6.35)	80
**Received antibiotics in the previous 3 months**									
Third-generation cephalosporin	11	2318	384	777		249	1541	4.81 (2.25 to 10.27)	89
Vancomycin	3	813	69	235		79	578	3.39 (2.21 to 5.20)	0
Quinolone	5	1242	105	477		55	765	2.99 (1.04 to 8.63)	79
Carbapenem	5	1156	68	405		49	751	2.85 (1.47 to 5.53)	42
Aminoglycoside	7	1444	151	485		235	959	2.84 (1.21 to 6.65)	83
Penicillin	9	1750	380	798		249	952	2.87 (1.10 to 7.47)	92
**Received antibiotic prophylaxis **	4	703	84	238		132	465	1.82 (1.16 to 2.86)	0
**Received any antibiotic **	13	2289	340	584		457	1705	3.58 (2.30 to 5.57)	60
**Received appropriate empirical antibiotic therapy **	5	803	102	192		463	611	0.29 (0.11 to 0.79)	65
**Exposed to invasive life support **									
Total parenteral nutrition	5	805	216	283		350	522	3.77 (1.35 to 10.56)	79
Continuous positive airway pressure	3	682	148	241		303	441	3.35 (0.54 to 20.61)	91
Mechanical ventilation	6	1098	137	432		271	666	3.29 (1.03 to 10.53)	83
Endotracheal intubation	8	1157	187	407		347	750	2.06 (1.22 to 3.49)	61
Central venous catheter	9	957	244	352		429	605	1.69 (1.00 to 2.85)	41

CI: confidence interval; EBSL: extended-spectrum β-lactamase-producing bacteria; NA: not applicable; OR: odds ratio.

^a^ Mantel–Haenszel random-effects.

^b^ Pre-infection hospitalization is the time of hospitalization to the time while patients with confirmed infection with extended-spectrum β-lactamase-producing Enterobacteriaceaeis, expressed as mean difference in days between positive and negative patients (standard deviation).

The risk of infection was higher for patients with co-existing bacteraemia (OR: 5.3; 95% CI: 3.64 to 7.72; *I^2^*: 38%; six studies), nosocomial infections (OR: 5.2; 95% CI: 2.23 to 12.07; *I^2^*: 92%; two studies), lower respiratory tract infections (OR: 5.0; 95% CI: 13.50 to 7.19; *I^2^*: 79%; four studies), sepsis (OR: 4.6 95% CI: 3.34 to 6.35; *I^2^*: 80%; 10 studies) or recurrent urinary tract infections (OR: 2.0; 95% CI: 1.61 to 2.43; *I^2^*: 90%; 11 studies; Table 2).

Antibiotics associated with risk of infection included third-generation cephalosporins (OR: 4.8; 95% CI: 2.25 to 10.27; *I^2^*: 89; 11 studies), vancomycin (OR: 3.4; 95% CI: 2.21 to 5.20; *I^2^*: 0%; three studies) and quinolones (OR: 3.0; 95% CI: 1.04 to 8.63, *I^2^*: 79; five studies). Five studies reported that appropriate initiation of empirical antibiotics was protective, showing a pooled OR of infection of 0.29 (95% CI: 0.11 to 0.79; *I^2^*: 65%; five studies; Table 2).

Exposure to continuous positive airway pressure therapy was not significantly associated with a risk of infection (OR: 3.4; 95% CI: 0.54 to 20.61; three studies). Other types of invasive life support were a risk, however. The OR for total parenteral nutrition was 3.8 (95% CI: 1.35 to 10.56; five studies). For mechanical ventilation the OR was 3.3 (95% CI: 1.03 to 10.53; six studies) and for endotracheal intubation 2.1 (95% CI: 1.22 to 3.49; eight studies). Central venous catheterization had an OR of 1.7 (95% CI: 1.00 to 2.85; nine studies; Table 2).

#### Treatment outcomes

Most specimens from patients with extended-spectrum β-lactamase-producing bacterial infection showed resistance to multiple antibiotics. The risk of antibiotic resistance was highest for extended-spectrum β-lactamase-positive patients treated with cephalosporins (OR: 70.5; 95% CI: 43.25 to 115.02; *I^2^*: 83%; 25 studies) and lowest with cotrimoxazole (OR: 1.8; 95% CI: 1.35 to 2.47; *I^2^*: 43%; 15 studies). The ORs for resistance to tetracyclines and nitrofurantoin were not statistically significant (Table 3).

**Table 3:** Pooled risk of antibiotic resistance to extended-spectrum β-lactamase-producing bacteria in specimens from children and young adults in South-East Asia and Western Pacific countries by antibiotic class, 2002–2018

Antibiotic class	No. of studies	Total no. of patients	ESBL-positive		ESBL-negative		Pooled OR (95% CI)^a^	*I^2^*, %
			Events	Total	Events	Total		
Cephalosporins	25	3444	1339	1483	632	1961	70.50 (43.25 to 115.02)	83
Monobactams	8	879	274	412	63	467	41.16 (14.05 to 120.55)	58
Penicillins	24	3148	1160	1304	1091	1844	19.41 (8.67 to 43.46)	86
Aminoglyclosides	25	3449	495	1452	276	1997	5.71 (3.42 to 9.54)	74
Combinations^b^	22	2993	706	1141	739	1852	4.37 (1.95 to 9.82)	91
Carbapenems	22	2940	79	1244	64	1696	3.99 (1.68 to 9.48)	0
Fluoroquinolones	25	3351	627	1439	607	1912	3.33 (2.14 to 5.17)	78
Cotrimoxazole	15	2346	547	868	755	1478	1.82 (1.35 to 2.47)	43
Tetracyclines	7	1447	355	619	357	828	1.58 (0.76 to 3.30)	81
Nitrofurantoin	3	1039	58	423	90	6	0.97 (0.64 to 1.46)	14

CI: confidence interval; EBSL: extended-spectrum β-lactamase-producing bacteria; OR: odds ratio.

^a^ Mantel–Haenszel random-effects.

^b^ Combinations: Ampicillin + sulbactam; ticarcillin + clavulanic acid; amoxicillin + clavulanate; cefoperazone + sulbactam; piperacillin + tazobactam; ceftazidime+ clavulanic acid.

**Fig. 2:**
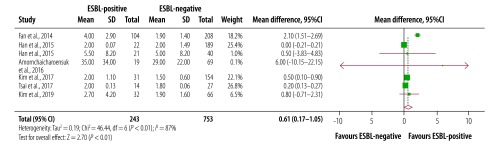
Duration of fever after antibiotic therapy among children and young adults with and without extended-spectrum β-lactamase-associated infection in South-East Asia and Western Pacific countries

CI: confidence interval; EBSL: extended-spectrum β-lactamase-producing bacteria; SD: standard deviation.

Note: We made inverse variance (IV) random-effects.

**Fig. 3:**
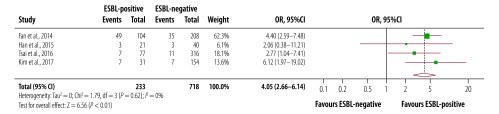
Persistent bacteraemia among children and young adults with and without extended-spectrum β-lactamase-associated infection in South-East Asia and Western Pacific countries

CI: confidence interval; EBSL: extended-spectrum β-lactamase-producing bacteria; OR: odds ratio.

Note: We made Mantel–Haenszel random-effects.

**Fig. 4:**
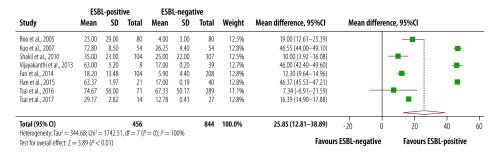
Length of hospital stay among children and young adults with and without extended-spectrum β-lactamase-associated infection in South-East Asia and Western Pacific countries

CI: confidence interval; EBSL: extended-spectrum β-lactamase-producing bacteria; SD: standard deviation.

Note: We made inverse variance (IV) random-effects.

The duration of fever was 0.61 days longer in patients with extended-spectrum β-lactamase-producing bacteria than patients without (95% CI: 0.18 to 0.72; *I^2^*: 92%; seven studies; Fig. 2). Pooling five studies we found that persistent bacteraemia was four times higher in patients positive for extended-spectrum β-lactamase-producing bacteria (95% CI: 2.66 to 6.14; *I^2^*: 0%; Fig. 3). Results from eight studies showed that the mean difference in length of hospital stay was 25.9 days (95% CI: 12.81 to 38.89; *I^2^*: 100%) for patients with extended-spectrum β-lactamase-associated infection than those without such infection (Fig. 4). Subgroup analysis showed that the mean length of hospital stay associated with infection was 29 days longer for patients who had recently been admitted to an intensive care unit care than the patients not receiving this care. Similar results were seen for invasive life support; the mean length of stay after central venous catheterization was 33 days longer than without catheterization.^59^

**Fig. 5:**
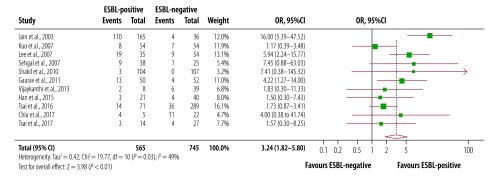
Mortality among children and young adults with and without extended-spectrum β-lactamase-associated infection in South-East Asia and Western Pacific countries

CI: confidence interval; EBSL: extended-spectrum β-lactamase-producing bacteria; OR: odds ratio.

Note: We made Mantel–Haenszel random-effects.

Eleven studies reported a pooled number of 188 deaths among 565 patients with extended-spectrum β-lactamase-associated infections compared with 86 deaths in 745 patients without these infections (OR: 3.2; 95% CI: 1.82 to 5.80; *I^2^:* 49%; Fig. 5). When analysed by subgroups, the risk of death for patients who had previously been admitted to the intensive care unit or exposed to central venous catheterization were not significant. However, the risk of death was higher among patients with sepsis (OR: 4.9 95% CI: 2.11 to 11.39; *I^2^*: 38%) than those without sepsis (OR: 2.3 95% CI: 1.19 to 4.26; *I^2^*: 35%;).^59^

We also looked at the ORs for neonates and non-neonates but the differences not statistically significant between these groups.^59^

#### Validity tests

None of the factors we analysed by meta-regression were contributors to between-study heterogeneity. In the Newcastle-Ottawa analysis of risk of bias, we found that 60% (24 out of 40) of studies scored high on risk of bias and 40% were low risk (Table 4; Table 5). Only four studies had clear statements about comparability and 10 about representativeness. The results from Egger’s regression test revealed that publication bias was significant (*P* < 0.001). Sensitivity analysis excluding small studies with samples less than 10 revealed that the funnel plots were consistently asymmetric (*P* < 0.001; available from the corresponding author). The sensitivity analysis showed that the data were not consistent with from the overall estimated ORs and similar trends were observed. This evaluation showed that a more restricted analysis of the data did not affect the magnitude, direction and the overall summary estimate.

**Table 4:** Risk of bias in case–control and cross-sectional studies included in the meta-analysis of extended-spectrum β-lactamase-associated infection among children and young adults in South-East Asia and Western Pacific countries, 2005–2018

Author	Selection					Comparability				Exposure	Total score^b^
	Representativeness of sample	Sample size	Non-respondents	Ascertainment of exposure (risk factor)		Different outcome groups are comparable; confounding factors are controlled^a^	Assessment of exposure or outcome	Same method of ascertainment for cases and controls		Non-response rate or statistical test	
Boo et al., 2005^22^	0	1	1	1		0	1	1		1	6
Kuo et al., 2007^26^	0	1	1	0		0	1	1		1	5
Gaurav et al., 2011^33^	0	1	1	0		0	1	1		1	5
Minami et al., 2012^36^^,c^	1	0	0	0		0	1	1		1	4
Fan et al. 2014^39^	0	1	1	1		0	1	1		1	6
Themphachana et al., 2014^40^^,c^	0	1	0	0		0	1	1		1	4
Young et al., 2014^41^^,c^	1	1	0	1		0	1	1		0	5
Zuo et al., 2014^42^^,c^	0	0	1	0		1	1	0		1	4
Sharma et al., 2016^48^^,c^	0	1	0	1		0	1	1		1	5
Tsai et al., 2017^55^	1	1	0	1		0	1	1		1	6
Chen et al., 2017^50^	1	1	0	0		0	1	1		1	5
Bunjoungmanee et al., 2018^56^	0	1	1	0		0	1	1		0	4
Kitagawa et al., 2018^57^	0	1	1	0		0	1	1		0	4

^a^ Subjects in different outcome groups are comparable, based on the study design or analysis.

^b^ Maximum score: 8.

^c^ Cross-sectional study.

Notes: We applied the Newcastle–Ottawa scale to assess risk of bias in non-randomized studies.^14^ Only studies scoring ≥ 5 and ≤ 8 were designated low risk of bias, ≥ 3 and ≤ 4 as moderate and ≤ 2 as high. We made Mantel-Haenszel radom-effects

**Table 5:** Risk of bias in cohort studies included in the meta-analysis of extended-spectrum β-lactamase-associated infection among children and young adults in South-East Asia and Western Pacific countries, 2002–2018

Author	Selection					Comparability				Exposure	Total score^b^
	Representativeness of the exposed cohort	Selection of the non-exposed cohort	Ascertainment of exposure	Demonstration that outcome of interest was not present at the start of study		Cohorts are comparable based on the design or analysis	Assessment of outcome^a^	Follow -up long enough for outcomes to occur		Adequacy of follow-up of cohorts	
Kim et al., 2002^20^	0	0	1	0		0	1	1		1	4
Jain et al., 2003^21^	0	0	1	0		0	1	1		1	4
Chiu et al., 2005^23^	1	0	1	0		0	1	1		1	5
Huang et al., 2007^24^	0	0	1	0		0	1	1		1	4
Jain & Mondal, 2007^25^	0	0	1	0		0	1	1		1	4
Lee et al., 2007^27^	1	0	1	0		0	1	1		1	5
Sehgal et al., 2007^28^	0	0	1	0		0	1	1		1	4
Bhattacharjee et al., 2008^29^	0	0	1	0		0	1	1		1	4
Anandan et al., 2009^30^	0	0	1	0		0	1	1		1	4
Kim et al., 2009^31^	0	0	1	0		0	1	1		1	4
Shakil et al., 2010^32^	1	0	1	0		0	1	1		1	5
Liu et al., 2011^34^	0	0	1	0		0	1	1		1	4
Wei et al., 2011^35^	0	0	1	0		0	1	1		1	4
Zheng et al., 2012^37^	0	0	1	0		0	1	1		1	4
Vijayakanthi et al., 2013^38^	0	0	1	0		0	1	1		1	5
Themphachana et al., 2014^40^	0	0	1	0		0	1	1		1	4
Duong et al., 2015^43^	0	0	1	0		0	1	1		1	4
Han et al., 2015^17^^,c^	0	0	1	0		0	1	1		1	4
Han et al., 2015^44^^,d^	0	0	1	0		0	1	1		1	4
Nisha et al., 2015^45^	0	0	1	0		1	1	1		1	5
Agarwal et al., 2016 ^46^	0	0	1	0		0	1	1		1	4
Amornchaicharoensuk, 2016^47^	0	0	1	0		1	1	1		1	5
He et al., 2017^51^	1	0	1	0		0	1	1		1	5
Kim et al., 2017^52^	1	0	1	0		0	1	1		1	5
Mandal et al., 2017^53^	0	0	1	0		1	1	1		0	4
Nisha et al., 2017^54^	0	0	1	0		0	1	1		1	4
Tsai et al., 2017^55^	1	0	0	0		0	1	1		1	4
Weerasinghe et al., 2018^58^	0	1	1	0		0	1	1		0	4

^a^ Subjects in different outcome groups are comparable, based on the study design or analysis. Confounding factors are controlled

^b^ Maximum score: 8.

^C^ Neutropoenia study

^d^ Urinary tract infection study.

Notes: We applied the Newcastle–Ottawa scale to assess risk of bias in non-randomized studies.^14^ Only studies scoring ≥ 5 and ≤ 8 were designated low risk of bias, ≥ 3 and ≤ 4 as moderate and ≤ 2 as high.

### Prevalence study

The overall pooled prevalence of extended-spectrum β-lactamase in the studies included the meta-analysis combined with surveillance reports was 25.3%. The pooled prevalence from the studies in the meta-analysis was 39% among the 31 studies conducted in hospital settings and 31% in the seven studies conducted in community settings (two studies were in both hospital and the community). 

**Fig. 6:**
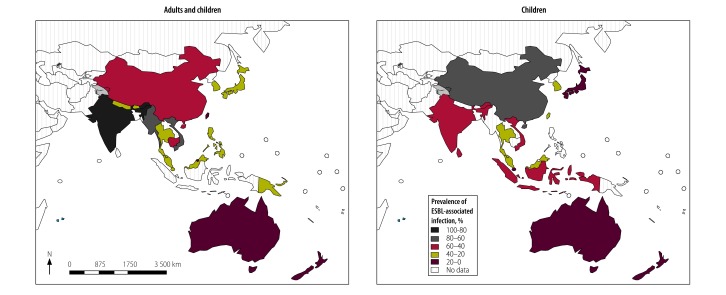
Map of prevalence of extended-spectrum β-lactamase-associated infection in South-East Asia and Western Pacific countries

Using data from other sources, we mapped population surveillance data from a total of 21 countries and areas in the South-East Asia and the Western Pacific Regions (Table 6). The pooled data from all available surveillance resources that included adults and children showed that India had the highest pooled prevalence (90.0%) and Australia the lowest (3.6%; numerators and denominators unavailable). The pooled data specifically for children, where available from surveillance resources and published data, showed similar results (Fig. 6).

**Table 6:** Pooled prevalence of overall population of extended-spectrum β-lactamase-associated infection from available surveillance data in 20 South-East Asia and Western Pacific countries or areas

Country or area	Data source^a^	Prevalence in children^b^ by data source			Prevalence in children and adults^c^ by data source		Pooled prevalence, %
		No. of people	No. (%) ESBL-positive		No. of people	No. (%) ESBL-positive	
Australia	SENTRY, 1998–1999^60^	NA	NA		660	8 (1.2)	3.6
	SMART, 2011^61^	80	2 (2.5)		80	2 (2.5)	
	CDDEP, 2011–2014^10^	NA	NA		NR	NR (4.5)	
	AURA, 2015^62^	NA	NA		NR	NR (6.0)	
Bhutan	CDDEP, 2011–2014^10^	NA	NA		NR	NR (29.5)	29.5
Brunei Darussalam	CDDEP, 2011–2014^10^	NA	NA		NR	NR (4.5)	4.5
Cambodia	CDDEP, 2011–2014^10^	NA	NA		NR	NR (49.5)	49.5
China	SENTRY, 1998–1999^61^	NA	NA		247	63 (25.5)	47.5
	CDDEP, 2011–2014^10^	NA	NA		NR	NR (69.5)	
China, Hong Kong Special Administrative Region	SENTRY, 1998–1999^61^	NA	NA		324	43 (13.3)	13.3
Taiwan, China	SENTRY, 1998–1999^61^	NA	NA		139	11 (7.9)	7.9
India	CDDEP, 2011–2014^10^	NA	NA		NR	NR (90.0)	90.0
Japan	SENTRY, 1998–1999^61^	NA	NA		272	18 (6.6)	10.6
	CDDEP, 2011–2014^10^	NA	NA		NR	NR (14.5)	
Malaysia	CDDEP, 2011–2014^10^	NA	NA		NR	NR (14.5)	14.5
Federated States of Micronesia (Federated States of)	CDDEP, 2011–2014^10^	NA	NA		NR	NR (69.5)	69.5
Myanmar	CDDEP, 2011–2014^10^	NA	NA		NR	NR (69.5)	69.5
Nepal	CDDEP, 2011–2014^10^	NA	NA		NR	NR (29.5)	29.5
New Zealand	CDDEP, 2011–2014^10^	NA	NA		NR	NR (4.5)	3.7
	ESR, 2016^63^	NR	NR (2.8)		NR	NR (2.8)	
Papua New Guinea	CDDEP, 2011–2014^10^	NA	NA		NR	NR (29.5)	29.5
Philippines	SENTRY, 1998–1999^61^	NA	NA		298	58 (19.5)	24.5
	CDDEP, 2011–2014^10^	NA	NA		NR	NR (29.5)	
Republic of Korea	CDDEP, 2011–2014^10^	NA	NA		NR	NR (29.5)	29.5
Singapore	SENTRY, 1998–1999^61^	NA	NA		153	31 (20.3)	20.3
Thailand	CDDEP, 2011–2014^10^	NA	NA		NR	NR (29.5)	29.5
Viet Nam	SMART, 2011^61^	38	15 (39.5)		38	15 (39.5)	54.5
	CDDEP, 2011–2014^10^	NA	NA		NR	NR (69.5)	

EBSL: extended-spectrum β-lactamase; NA: not applicable; NR: not reported.

^a^ Data sources: AURA: Antimicrobial Use and Resistance in Australia Surveillance System; CDDEP: Center for Disease Dynamics, Economics & Policy; ESR: Institute of Environmental Science and Research Surveillance System in New Zealand; SENTRY: Antimicrobial Surveillance Program by JMI Laboratories; SMART: Study for Monitoring Antimicrobial Resistance Trends.

^b^ Ages 0–21 years.

^c^ Ages not specified.

Notes: We searched the published and grey literature for surveillance data from all Member States and areas in the World Health Organization South-East Asia and Western Pacific Regions. No data were available for: Bangladesh, Cook Islands, Democratic People's Republic of Korea, Fiji, Indonesia, Kiribati, Lao People's Democratic Republic, Maldives, Marshall Islands, Mongolia, Nauru, Niue, Palau, Samoa, Solomon Islands, Timor-Leste, Tonga, Tuvalu and Vanuatu.

ESBL: extended-spectrum β-lactamase.

Notes: We pooled data from a search of the published and grey literature for surveillance data from all Member States and areas in the World Health Organization South-East Asia and Western Pacific Regions. The map for adults and children includes data from Australia; Bhutan; Brunei Darussalam; Cambodia; Taiwan, China; China, Hong Kong Special Administrative Region; India; Indonesia; Japan; Federated States of Micronesia (Federated States of); Myanmar; Nepal; New Zealand; Papua New Guinea; Philippines; Republic of Korea; Singapore; Thailand and Viet Nam. The map for children only (ages 0–21 years) includes data from Australia (2.5%); China (54.3%); Taiwan, China (28.7%); India (45.9%); Indonesia (39.0%); Japan (9.0%); New Zealand (2.8%); Republic of Korea (23.7%); Singapore (4.0%); Sri Lanka (36.0%); Thailand (21.7%); and Viet Nam (39.5%).

## Discussion

This study revealed that the average combined prevalence of infection with extended-spectrum β-lactamase-producing bacteria among children in South-East Asia and the Western Pacific is high. Risk factors for infection included recent intensive care unit admission, hospitalization, surgery or antibiotic exposure, and co-existing bacteraemia, nosocomial infections, lower respiratory tract infections, sepsis or recurrent urinary tract infections. Infection was associated with higher mortality, higher morbidity and longer hospitalization.

The prevalence of infection we found in South-East Asia and the Western Pacific countries are similar to those reported from other surveillance systems worldwide,^64^although many locations do not report data for children. A review of worldwide trends in extended-spectrum β-lactamase-associated infection reported higher prevalence in Asia, Latin America and the Middle East (from 28 to 40%) compared with other, higher-income areas (from 8 to 12%).^64^

As many of the studies we found were hospital-based our results support the need for resources and policies for control of nosocomial infection. A recently published modelling study showed that antibiotic use in hospital is a major driver for antimicrobial resistance in human infection compared with animal and environmental antibiotic exposures.^65^ Although infection control and hygiene may be sub-optimal in the countries we studied, infection control is easier to manage within health-care institutions than in other unstructured systems such as animal husbandry and the environment. Without proper control of antimicrobial resistance in hospitals, patients can disseminate antibiotic residues and resistance genes to the community and environment. This still highlights the importance of hospital-based stewardship for controlling antibiotic use and how this stewardship can reduce the risk of developing multidrug resistant organisms.^66^ At the same time, the rising community prevalence of extended-spectrum β-lactamase-associated infection provides evidence for expanding prevention to other settings.^3^

Our meta-analysis showed that recent medical care, including intensive care unit stays, hospitalization, surgery and antibiotic therapy, was associated with increased risk of infection. These results suggest that children may acquire such infections during health care, especially when undergoing invasive procedures. Specifically, children who had exposure to third-generation cephalosporins, carbapenems and fluoroquinolones had three to four times greater risk for extended-spectrum β-lactamase-associated infection, which is similar to previous reports.^26^^,^^67^^–^^71^ As these antibiotics are primarily used for treating severe infections, their use may be a marker for disease severity rather than a direct contributor to developing resistance. Nevertheless, if excessive fluoroquinolone use does contribute to emergence of resistant bacteria this adds another reason to avoid the unnecessary use of these broad-spectrum antibiotics in children.

Coexisting illnesses, including bacteraemia, nosocomial infection, lower respiratory tract infections, sepsis and recurrent urinary tract infections, were associated with increased risk of infection. These co-morbidities could be risk factors for use of invasive treatments such as a central venous catheterization, mechanical ventilation, intravenous nutrition or increased risk of interactions with health-care settings. In a two-centre case–control study of risk factors for infection with extended-spectrum β-lactamase-producers in children, multivariable analysis identified sepsis and neurological illnesses as potential risk factors, which supports our findings.^72^ Previously published studies among both young adults and children found that prolonged hospital stay or prolonged use of invasive medical devices were associated with infection by, or being colonized with, extended-spectrum β-lactamase-producing bacteria,^26^^,^^69^^,^^71^ which is consistent with our findings.

Recent surgery and antibiotic prophylaxis were associated with extended-spectrum β-lactamase infection in our study. Others have shown that surgical antibiotic prophylaxis increases the risk for antimicrobial resistance and acquisition of infection.^73^ One study from Switzerland found that half of all surgical ward prescriptions (680 out of 1270) were inappropriate.^74^ Antibiotic stewardship programmes have been shown to improve surgical antibiotic prophylaxis and treatment of surgical site infections.^75^

Our study found that initiation of appropriate empirical antibiotics was protective against extended-spectrum β-lactamase-associated infection, indicating the importance of thoughtful selection of antibiotics. The details of this finding warrant further study. The risk is especially high for critically ill patients requiring surgery or intensive care and who need antibiotics urgently before susceptibility has been established but who are also at increased risk for drug-resistant infections. Therefore, antibiotic stewardship programmes and guidelines in health-care facilities fill an important function. Furthermore, as studies in Asia have shown a high prevalence of easy access to unsupervised antibiotics within the community, more attention is needed to improving appropriate antibiotic use through training, education, policy and regulation outside of hospitals.^76^

Children infected with extended-spectrum β-lactamase-producers had significantly longer length of hospital stays (26 days) and required more intensive care unit days (29 days) than those without such infection. This leads to higher health-care costs,^77^ in addition to the costs to society in terms of family and community pressures and lost productivity. At the same time, prolonging intensive care unit and hospital stays increases the risk of further acquisition and transmission of drug resistance.

Mortality and persistent bacteraemia were three to four times higher for patients infected with extended-spectrum β-lactamase-associated infections than those without. This adds to the economic and social burden of these infections. Based on our meta-regression, the study location, study design, patient’s diagnosis, sex or intensive care unit stay did not influence mortality. This implies that worse outcomes may be directly attributable to the presence of extended-spectrum β-lactamase-associated infection. The severity of the diseases associated with these infections might also contribute to mortality risk, as the patients diagnosed with sepsis had higher risk of mortality than those without sepsis. However, we were unable to determine for each study whether other factors may have influenced outcomes because comprehensive information was not available.

One of the strengths of our study was the comprehensive data collection strategy, which provided a high sample size and study power. Second, two different tools were used to assess for bias, which, together with risk factor and outcomes sensitivity analysis, strengthened the study’s validity and reliability. Third, we assessed previous antibiotic history with different antibiotic categories, providing a detailed insight into the link between antibiotic use and resistance. Fourth, we also conducted meta-regression to determine if other factors might have influenced treatment outcomes. This established association between patients’ mortality, length of stay and extended-spectrum β-lactamase infections.

There were several limitations to this study. The distribution of studies between locations was not uniform. Of the 48 Member States and areas in the South-East Asia and the Western Pacific Regions, we were able to find and extract data for the meta-analysis from 12 countries. For prevalence estimates we added surveillance data from 10 other countries and areas but we found data on 0–21-years-olds for only three countries with available paediatric data, which might underestimate the real situation among children. Moreover, although we made subgroup analyses, most of the pooled prevalence from selected studies were from hospital settings. Most of the surveillance sources reported only prevalence, without denominators and numerators. Nevertheless, the study provides a rough indication of the extent of extended-spectrum β-lactamase-associated infection and highlights the need for establishment of surveillance systems in these Regions. We can expect that within large Regions, rates of infection are unlikely to be homogenous, particularly where there are large urban and rural disparities. Among 40 studies, only seven were community based. This might have underestimated antibiotic resistance in the community. With the rising concern for community-acquired infections and reports of increased rates of faecal colonization with extended-spectrum β-lactamase-producing bacteria in healthy children, risk factors might not only arise from hospital influences but also from community exposure and international travel.^78^^–^^80^ Because of limited information in the articles, we are unable to determine whether longer hospitalization increased the risk of infections or vice versa. Both situations are likely and further studies are needed to clarify the associations.

Another limitation we faced was the lack of laboratory standardization for the identification of the extended-spectrum β-lactamase-producer phenotypes. Quality and standardization may vary between laboratories, although most followed Clinical and Laboratory Standards Institute guidelines. Sensitivity analyses found that use of different laboratory guidelines or test methods or the study year did not affect our results. All studies used phenotypic methods, as opposed to the gold standard through genotyping, with the majority using agar double-disk diffusion test, while a few studies used the Vitek® system (bioMérieux, Marcy l’Etoile, France). Thus, detection rates could be underestimated.

We hope this study will provide important information for policy-makers who need to allocate resources to improve surveillance, monitor treatment outcomes, improve infection control in intensive care unit and surgery wards and develop policies for the use of empirical and prophylactic antibiotics. Knowledge of resistance rates can guide treatment recommendations. Countries without established antibiotic stewardship programmes should prioritize these activities, along with public education programmes. With very high burden of neonatal sepsis 0.42 million (39%) of the total 1.09 million deaths related with sepsis in these Regions,^81^ scaling up strategies to prevent infection and encourage appropriate treatment for this vulnerable group is needed. More studies are also needed to measure the impact of antimicrobial resistance in children.
