# Corrigendum

**DOI:** 10.2471/BLT.19.110819

**Published:** 2019-08-01

In: Hu YJ, Ogyu A, Cowling BJ. Fukuda K, Pang HH. Available evidence of antibiotic resistance from extended-spectrum β-lactamase-producing Enterobacteriaceae in paediatric patients in 20 countries: a systematic review and meta-analysis. Bull World Health Organ. 2019 Jul 1;97(7):486–501B

on page 491, the fourth sentence should read as follows:

The sensitivity analysis showed that the data were consistent with from the overall estimated ORs and similar trends were observed.

